# Multi-Region Radiomic Analysis Based on Multi-Sequence MRI Can Preoperatively Predict Microvascular Invasion in Hepatocellular Carcinoma

**DOI:** 10.3389/fonc.2022.818681

**Published:** 2022-04-27

**Authors:** Lanmei Gao, Meilian Xiong, Xiaojie Chen, Zewen Han, Chuan Yan, Rongping Ye, Lili Zhou, Yueming Li

**Affiliations:** ^1^ Department of Radiology, The First Affiliated Hospital of Fujian Medical University, Fuzhou, China; ^2^ The School of Medical Technology and Engineering, Fujian Medical University, Fuzhou, Fujian, China; ^3^ Key Laboratory of Radiation Biology (Fujian Medical University), Fujian Province University, Fuzhou, Fujian, China

**Keywords:** hepatocellular carcinoma, microvascular invasion, radiomics, machine learning, magnetic resonance imaging, nomogram

## Abstract

**Objectives:**

Microvascular invasion (MVI) affects the postoperative prognosis in hepatocellular carcinoma (HCC) patients; however, there remains a lack of reliable and effective tools for preoperative prediction of MVI. Radiomics has shown great potential in providing valuable information for tumor pathophysiology. We constructed and validated radiomics models with and without clinico-radiological factors to predict MVI.

**Methods:**

One hundred and fifteen patients with pathologically confirmed HCC (training set: n = 80; validation set: n = 35) who underwent preoperative MRI were retrospectively recruited. Radiomics models based on multi-sequence MRI across various regions (including intratumoral and/or peritumoral areas) were built using four classification algorithms. A clinico-radiological model was constructed individually and combined with a radiomics model to generate a fusion model by multivariable logistic regression.

**Results:**

Among the radiomics models, the model based on T2WI and arterial phase (T2WI-AP model) in the volume of the liver–HCC interface (VOI_interface_) exhibited the best predictive power, with AUCs of 0.866 in the training group and 0.855 in the validation group. The clinico-radiological model exhibited good efficacy (AUC: 0.819 and 0.717, respectively). The fusion model showed excellent predictive ability (AUC: 0.915 and 0.868, respectively), outperforming both the clinico-radiological and the T2WI-AP models in the training and validation sets.

**Conclusion:**

The fusion model of multi-region radiomics achieves an enhanced prediction of the individualized risk estimation of MVI in HCC patients. This may be a beneficial tool for clinicians to improve decision-making in personalized medicine.

## Introduction

Hepatocellular carcinoma (HCC) comprises 75–85% of primary liver cancers, making it the sixth most prevalent cancer and the third leading cause of global cancer mortality in 2020 (1). While hepatectomy and liver transplantation are potentially curative treatments for HCC (2), recurrence after surgery is common (3). The five-year recurrence ratio of HCC after hepatic resection is almost 70%, and around 10–15% after liver transplantation (4). Previous studies have shown that microvascular invasion (MVI) is an independent risk factor for postoperative recurrence and poor prognosis (5, 6). In contrast to macrovascular invasion in HCC patients, which can be detected through preoperative imaging (7), MVI is mainly identified by postoperative pathological examination. Thus, preoperative and noninvasive prediction of MVI may significantly impact clinical decision making, individual comprehensive therapy and prognosis assessment.

Many studies have been conducted to identify factors related to MVI, namely, clinical indicators and imaging characteristics. To date, laboratory biomarkers of MVI include alpha-fetoprotein (AFP), lectin-reactive AFP, prothrombin induced by vitamin K absence-II and other serum markers (8, 9). However, the efficacy of these biomarkers has varied among studies. For instance, the prediction performance of serum AFP was found to be unsatisfactory due to low specificity and sensitivity (8). Some radiological features, like peritumoral enhancement on arterial phase (AP), irregular rim-like arterial phase hyperenhancement, peritumoral hypointensity on hepatobiliary phase (HBP), and non-smooth tumor margin, have been hailed as radiologic hallmarks for predicting MVI, but lack consensus among studies (10–12). Furthermore, these qualitative features are liable to suffer from the personal bias of radiologists, thus introducing inter-observer variability. Therefore, preoperative prediction of MVI requires a more reliable and repeatable tool.

Radiomics is defined as the automated quantification of the radiological phenotype using data-characterization algorithms (13, 14). It is a vital imaging technology useful for differential diagnoses, assessment of therapeutic responses, prognosis prediction, etc., thereby providing valuable information for personalized medicine (13, 15, 16). Radiomic strategies have shown great predictive potential by incorporating radiological features related to various diseases and clinical and/or pathological factors into a single fusion model (17, 18). Recent studies have shown the clinical utility of radiomics based on computed tomography (CT) or magnetic resonance imaging (MRI), specifically for predicting MVI in HCC patients before surgery (19, 20). In addition to the intratumoral area, the peritumoral region, which contains complementary data outside the HCC volume, where MVI may still occur, has been studied (20–22). To the best of our knowledge, to-date there have been only two studies, which evaluated CT radiomics at the tumor–liver interface for predicting MVI and HCC recurrence (23, 24).

In this study, we not only focused on intratumoral and peritumoral areas but also the HCC–liver interface using multi-sequence MRI radiomics. Various radiomics models were constructed and validated based on diverse machine learning algorithms. Additionally, we constructed a fusion model based on a radiomics model and the clinico-radiological preoperative predictors of MVI.

## Materials and Methods

### Patients

**Figure 1:**
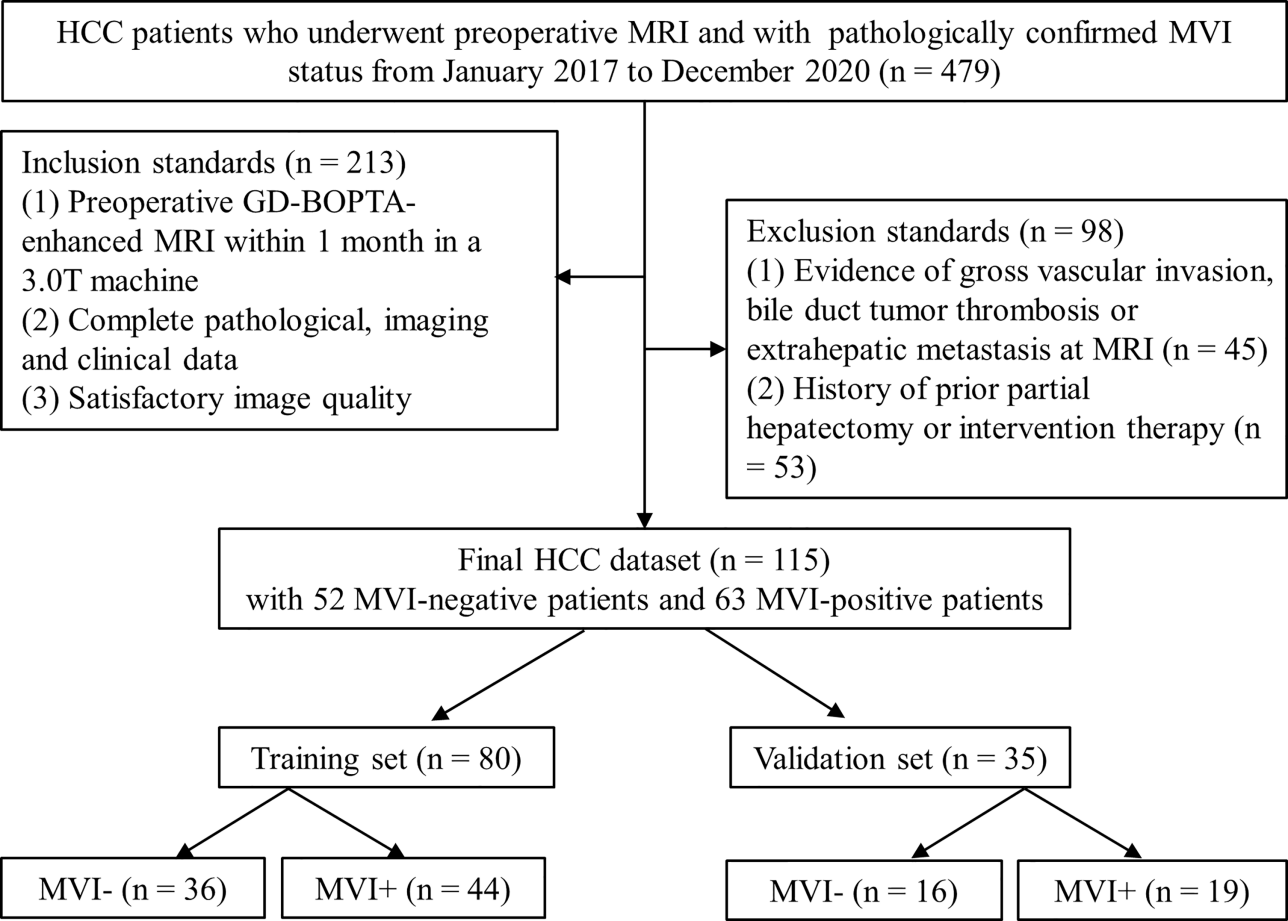
Flowchart of patients enrolled in the study.

This retrospective study was approved by the ethics committee of our hospital and the requirement for patient informed consent was waived. Between January 2017 and December 2020, 479 HCC patients who underwent preoperative MRI and with pathologically confirmed MVI-positive (MVI+) or MVI-negative (MVI−) were identified. The final cohort consisted of 115 consecutive patients (99 men and 16 women; 57.2 ± 10.9 years) who met the inclusion/exclusion criteria (**Figure 1**). The inclusion standards included (1) preoperative gadobenate dimeglumine (GD-BOPTA) enhanced MRI within 1 month in a 3.0 T machine (2); complete pathological, imaging, and clinical data record; and (3) satisfactory image quality. The exclusion criteria included (1) evidence of gross vascular invasion, bile duct tumor thrombosis or extrahepatic metastasis at MRI, or (2) history of prior partial hepatectomy or intervention therapy. The largest tumor was studied when the patients had more than one HCC lesion. Patients were randomly allocated into training cohort (*n* = 80) and validation cohort (*n* = 35) at a ratio of 7:3.

### Laboratory Tests and Histology

Clinical data collected before surgery included age, gender, hepatitis B and C immunology, AFP level, alanine aminotransferase, aspartate aminotransferase, r-glutamyltransferase, serum albumin, platelet count, prothrombin time, international normalized ratio, and total bilirubin. Hepatic virus infection, cirrhosis, and number of histologic tumors were also included in this study. The MVI status was assessed by two experienced abdominal pathologists postoperatively. MVI was defined as the presence of a tumor within a vascular space lined by the endothelium (observed by microscopy), mostly in the portal vein, hepatic vein, or a large capsular vessel of the surrounding hepatic tissue (21, 25). Evaluations were discussed and classified by consensus.

### MRI Protocol

All MRI were examined in a 3.0 T MRI machine (Magnetom Verio; Siemens Healthcare). The standard protocol consisted of the following sequences: transverse T2-weighted imaging (T2WI) with fat suppression, diffusion-weighted imaging (DWI), in-phase and opposed-phase T1-weighted imaging (T1WI), pre-contrast three-dimensional volumetric-interpolated breath-hold T1WI, and T1WI after contrast medium injection (AP, 20–30 s; portal venous phase: PVP, 60–70 s; delayed phase: DP, 2–3 min; HBP, 90 min). The detailed parameters are provided in **Table S1**.

### Radiological Evaluation

All MR images were independently reviewed by two radiologists with 15 and 3 years of MR experience, respectively, who were blinded to the clinical and pathological data. A final consensus was achieved after discussion if any disagreement existed. The following radiological characteristics of HCC were evaluated: i) maximum tumor length; ii) tumor margin (26); iii) tumor capsule (27); iv) non-peripheral washout (28); v) peritumoral arterial enhancement (26); vi) mosaic architecture (28); vii) tumor hypointensity on HBP (10); and viii) peritumoral hypointensity on HBP (29).

### Clinico-Radiological Model

The clinico-radiological model was developed based on MVI risk predictors by univariate and multivariate logistic regression analyses. In the training dataset, the single factor was evaluated by the univariate analysis and multivariate analysis included the variables with p-value inferior to 0.10 at univariate analysis. Those significant factors identified by multivariate analysis were entered into the clinico-radiological model as the risk predictors of the discrimination of MVI existence. The diagnostic capacity of the clinico-radiological model was further assessed in the validation dataset.

### Radiomics Analysis

**Figure 2:**
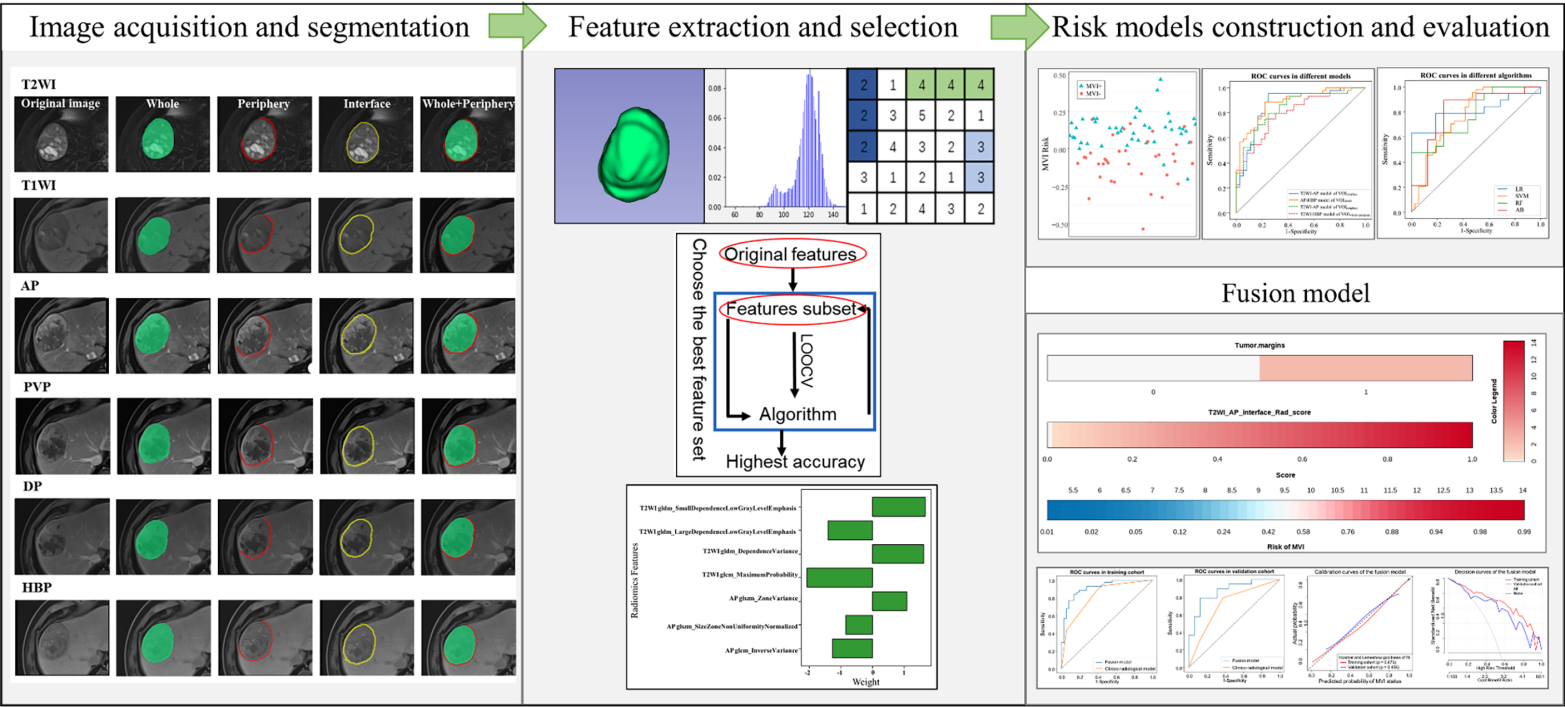
Workflow of radiomics analysis.

**Figure 3:**
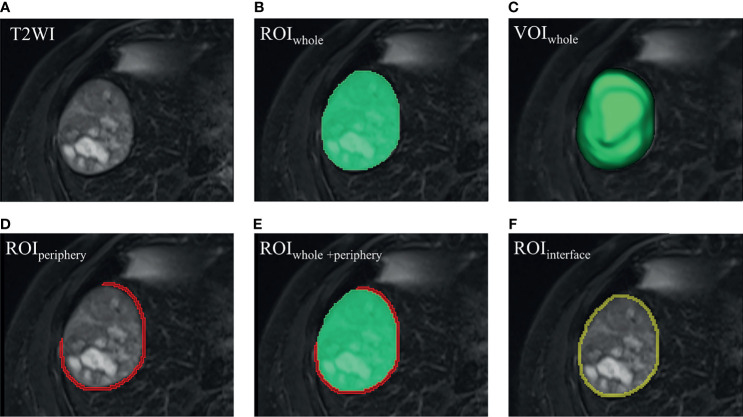
Visualized segmentation images. **(A)** HCC lesion in T2WI. **(B)** ROI_whole_ (green) was manually delineated slice by slice. **(C)** VOI_whole_ (green) was automatically constructed, covering the whole tumor. **(D)** On the bases of VOI_whole_, VOI_periphery_ was made hollow by replacing VOI_whole_ with a peritumoral 5-mm-thickness zone, and the covering part was erased when encountering liver margin, gall bladder, or large vessels (like the inferior vena cava). **(E)** VOI_whole + periphery_ was generated from the combination of VOI_whole_ and VOI_periphery_. **(F)** VOI_interface_ (yellow) was made by volume of 2-pixel automated dilation based on VOI_whole_ subtracting volume of 2-pixel shrinkage based on VOI_whole_, termed the liver–tumor interface. Please note that one pixel is around 1.2 mm, and then 4-pixel-wide band is approximately 5 mm. **(D–F)** 2-dimensional view of the VOI_periphery_, VOI_whole + periphery_ and VOI_interface_, respectively. ROI, region of interest. VOI, volume of interest.

Radiomics analysis workflow included image segmentation, feature extraction and selection, and model development and validation (**Figure 2**). First, a bias field correction in each sequence was performed using the N4ITK algorithm to remove field heterogeneity from the image. The volumes of interest (VOIs) were then manually delineated on T2WI, pre-contrast T1WI, AP, PVP, DP, and HBP, covering the whole tumor (i.e., VOI_whole_) by an abdominal radiologist (4 years of experience) using 3D Slicer software (https://www.slicer.org/). The peritumoral 5-mm-thickness zone (namely, VOI_periphery_) was then outlined to further explore the tumor periphery automatically. Meanwhile, VOI_whole + periphery_ was generated from the combination of VOI_whole_ and VOI_periphery_, and VOI_interface_ (a 5mm wide band at the liver–tumor interface) was made by SimpleITK (https://simpleitk.org/). Specific segmentation procedures and representative images are shown in **Figure 3**. Additionally, the MR images of 30 patients were randomly selected for re-segmentation one month later by a second abdominal radiologist (3 years of experience). The dice coefficients were used to compare segmented VOIs. The value of a dice coefficient ranges from 0, indicating no spatial overlap between two sets of binary segmentation results, to 1, indicating complete overlap.

Image normalization and spatial resampling were performed before the feature extraction to enable the normalization of image intensity values and allow acquisition of isotropic voxels, respectively (30, 31). In total, a set of 107 radiomic features were extracted using the open-source package PyRadiomics (version 3.0.1, https://www.radiomics.io/pyradiomics.html). Radiomic features were categorized into three types: shape-based characteristics, first-order statistics, and textural features. The detailed definitions of each feature can be found in the online PyRadiomics documentation (https://pyradiomics.readthedocs.io/en/latest/index.html). Next, the extracted radiomic features were transformed using Z-score normalization. Recursive feature elimination (RFE) with the leave-one-out cross-validation method was used for feature selection. By recursively removing the weakest attributes, dependencies and collinearity were eliminated.

In this research, four classification algorithms were used, namely, Logistic Regression (LR), Support Vector Classifier (SVC), Random Forest Classifier, and AdaBoost, implemented in the scikit-learn packages (version 0.24.1, https://scikit-learn.org/stable/index.html). Different algorithms wrapped by RFE were used to construct the radiomics model. All radiomic models were established on the basis of diverse sequences and various VOIs. Subsequently, the best sequences and VOI combinations were determined according to the AUC values. The multi-sequence models were based on single-sequence models that showed discriminative power AUCs greater than 0.75 in both the training and validation sets. The predictive ability of the evaluated models was then measured by the area under the receiver operating characteristic (ROC) curve (AUC). Accuracy, sensitivity and specificity were also assessed in both training and validation sets. The output values of the model were used as the radiomics signature.

### Fusion Model

The significant clinico-radiological factors identified by univariate analysis and the best signatures obtained from radiomic analysis (highest AUC or accuracy) were entered into multivariate logistic regression analysis. Those significant factors identified at multivariate analysis (*p <*0.05) were retained in the fusion model, and a nomogram was used as a graphical representation using the “rms” package. The discrimination efficacy was evaluated by AUC and the AUC values of different models were compared using the DeLong test. Additionally, the integrated discrimination improvement (IDI) was used to evaluate the improvement in average sensitivity of the fusion model without sacrificing average specificity relative to other models. Moreover, calibration plots and the Hosmer–Lemeshow test were used to describe the agreement between nomogram prediction and actual MVI. Additionally, decision curve analysis was performed to assess the clinical usefulness of the fusion model.

### Statistical Analyses

Continuous variables were analyzed using the Student *t*-test, and categorical variables were compared using a chi-square test or Fisher exact test for significant differences in the training and validation cohorts, as appropriate. The intraclass correlation coefficient (ICC) was calculated to verify the stability of the radiomic features. The correlations between selected features in the radiomics model in VOI_interface_ and tumor margin was assessed by the Spearman test. Statistical analysis was performed using the R software (version 4.0.2, http://www.r-project.org). Two-sided *p <*0.05 was considered statistically significant.

## Results

### Clinico-Radiological Characteristics and Predictive Performance

There were no statistically significant discriminative clinical or radiological factors identified between the training and validation cohorts (*p* = 0.242–1.000), as shown in **Tables 1**, **2**. However, univariate analysis identified that four radiologic factors (tumor margin, tumor capsule, peritumoral arterial enhancement, and peritumoral hypointensity on HBP) and one clinical variable (AFP) were significantly related to MVI in the training cohort (*p <*0.05). At the multivariate analysis, non-smooth margin (adjusted OR = 10.689, 95%CI = 3.397–40.052) and peritumoral hypointensity on HBP (adjusted OR = 6.007, 95%CI = 1.713–26.272) were identified as independent risk factors. Therefore, the clinico-radiological model for MVI prediction included these factors. The AUC (95% CI), accuracy, sensitivity, and specificity were 0.819 (0.732–0.905), 0.763, 0.886, and 0.611, respectively, in the training dataset with a cut-off of 0.552, and 0.717 (0.551–0.883), 0.714, 0.789, and 0.625 in the validation dataset, respectively.

**Table 1:** Clinical characteristics in the training and validation cohorts.

Clinical Variables	Training cohort (n = 80)				Validation cohort (n = 35)			*p_inter_* ^§^
	MVI− (n = 36)	MVI+ (n = 44)	OR (95% CI)^†^	*p_intra_* ^‡^	MVI− (n = 16)	MVI+ (n = 19)	*p_intra_* ^‡^	
Age, years^*^	59.2 (11.8)	58.3 (13.1)	0.995 (0.96–1.031)	0.770	57.9 (7.5)	59.2 (11.4)	0.705	0.972
Gender				0.499			0.187	0.562
Female	3 (8.3)	7 (15.9)	1.000		1 (6.2)	5 (26.3)		
Male	33 (91.7)	37 (84.1)	0.481 (0.115–2.011)		15 (93.8)	14 (73.7)		
AFP				0.044			0.167	1.000
≤400 ng/ml	29 (80.6)	25 (56.8)	1.000		13 (81.2)	11 (57.9)		
>400 ng/ml	7 (19.4)	19 (43.2)	3.149 (1.137–8.718)		3 (18.8)	8 (42.1)		
PLT				0.195			1.000	0.494
≤125 × 10^9^/L	6 (16.7)	14 (31.8)	1.000		3 (18.8)	3 (15.8)		
>125 × 10^9^/L	30 (83.3)	30 (68.2)	0.429 (0.145–1.265)		13 (81.2)	16 (84.2)		
PT				0.358			0.094	0.331
≤13 s	28 (77.8)	29 (65.9)	1.000		7 (43.8)	14 (73.7)		
>13 s	8 (22.2)	15 (34.1)	1.81 (0.664–4.936)		9 (56.2)	5 (26.3)		
INR				0.795			0.723	0.865
≤1.0	8 (22.2)	12 (27.3)	1.000		4 (25.0)	6 (31.6)		
>1.0	28 (77.8)	32 (72.7)	0.762 (0.272–2.131)		12 (75.0)	13 (68.4)		
TBIL				0.279			0.245	1.000
≤20.5 μmol/L	30 (83.3)	31 (70.5)	1.000		10 (62.5)	16 (84.2)		
>20.5 μmol/L	6 (16.7)	13 (29.5)	2.097 (0.705–6.235)		6 (37.5)	3 (15.8)		
ALB				0.428			0.315	0.784
≤40 g/L	18 (50.0)	17 (38.6)	1.000		6 (37.5)	11 (57.9)		
>40 g/L	18 (50.0)	27 (61.4)	1.588 (0.651–3.874)		10 (62.5)	8 (42.1)		
GGT				0.472			1.000	0.267
≤60 U/L	21 (58.3)	21 (47.7)	1.000		11 (68.8)	12 (63.2)		
>60 U/L	15 (41.7)	23 (52.3)	1.533 (0.631–3.727)		5 (31.2)	7 (36.8)		
ALT				1.000			1.000	0.242
≤50 U/L	27 (75.0)	32 (72.7)	1.000		14 (87.5)	16 (84.2)		
>50 U/L	9 (25.0)	12 (27.3)	1.125 (0.412–3.072)		2 (12.5)	3 (15.8)		
AST				1.000			0.047	0.343
≤40 U/L	24 (66.7)	29 (65.9)	1.000		15 (93.8)	12 (63.2)		
>40 U/L	12 (33.3)	15 (34.1)	1.034 (0.407–2.627)		1 (6.2)	7 (36.8)		
Hepatic virus infection				0.694			0.608	0.396
Absent	6 (16.7)	10 (22.7)	1.000		1 (6.2)	3 (15.8)		
Present (HBV/HCV)	30 (83.3)	34 (77.3)	0.68 (0.221–2.094)		15 (93.8)	16 (84.2)		
Cirrhosis				1.000			0.071	0.842
Absent	12 (33.3)	14 (31.8)	1.000		2 (12.5)	8 (42.1)		
Present	24 (66.7)	30 (68.2)	1.071 (0.419–2.741)		14 (87.5)	11 (57.9)		
Number of tumors				1.000			1.000	1.000
Solitary	32 (88.9)	38 (86.4)	1.000		14 (87.5)	17 (89.5)		
Multiple	4 (11.1)	6 (13.6)	1.263 (0.328–4.871)		2 (12.5)	2 (10.5)		

AFP, serum alpha-fetoprotein; PLT, platelet count; PT, prothrombin time; INR, international normalized ratio; TBIL, total bilirubin; ALB, serum albumin; GGT, r-glutamyltransferase; ALT, alanine aminotransferase; AST, aspartate aminotransferase; HBV, hepatitis B virus; HCV, hepatitis C virus. Except otherwise noted, data are numbers of patients, with the percentage in parentheses. P-value with Chi-square test or Fisher exact test for categorical variables and Student t-test for numeric variables. ^*^Data are means, with standard deviations in parentheses. ^†^Odds ratio (OR) with univariate test. ^‡^p_Intra_: p-value between the MVI+ and MVI− groups. ^§^p_Inter_: p-value between the training and validation cohorts.

**Table 2:** Radiological features in the training and validation cohorts.

Radiological variables	Training cohort (n = 80)				Validation cohort (n = 35)			*p_inter_* ^‡^
	MVI− (n = 36)	MVI+ (n = 44)	OR (95% CI)^*^	*p_intra_* ^†^	MVI− (n = 16)	MVI+ (n = 19)	*p_intra_* ^†^	
Maximum tumor diameter				0.223			0.002	0.386
≤5 cm	23 (63.9)	21 (47.7)	1.000		15 (93.8)	8 (42.1)		
>5 cm	13 (36.1)	23 (52.3)	1.938 (0.787–4.773)		1 (6.2)	11 (57.9)		
Tumor margin				<0.001			0.018	0.666
Smooth	22 (61.1)	5 (11.4)	1.000		10 (62.5)	4 (21.1)		
Non-smooth	14 (38.9)	39 (88.6)	12.257 (3.892–38.598)		6 (37.5)	15 (78.9)		
Nonperipheral washout				0.333			0.415	0.449
Absent	6 (16.7)	4 (9.1)	1.000		2 (12.5)	5 (26.3)		
Present	30 (83.3)	40 (90.9)	2 (0.518–7.721)		14 (87.5)	14 (73.7)		
Peritumoral arterial enhancement				0.021			0.071	0.947
Absent	30 (83.3)	25 (56.8)	1.000		14 (87.5)	11 (57.9)		
Present	6 (16.7)	19 (43.2)	3.8 (1.316–10.971)		2 (12.5)	8 (42.1)		
Tumor capsule				0.001			0.448	0.663
Complete	18 (50.0)	5 (11.4)	1.000		2 (12.5)	6 (31.6)		
Incomplete	11 (30.6)	23 (52.3)	7.527 (2.214–25.597)		9 (56.2)	9 (47.4)		
Absent	7 (19.4)	16 (36.4)	8.229 (2.175–31.133)		5 (31.2)	4 (21.1)		
Tumor hypointensity on HBP				0.401			1.000	0.674
Absent	4 (11.1)	2 (4.5)	1.000		0 (0.0)	1 (5.3)		
Present	32 (88.9)	42 (95.5)	2.625 (0.452–15.236)		16 (100.0)	18 (94.7)		
Peritumoral hypointensity on HBP				0.001			0.244	0.489
Absent	32 (88.9)	23 (52.3)	1.000		14 (87.5)	13 (68.4)		
Present	4 (11.1)	21 (47.7)	7.304 (2.209–24.154)		2 (12.5)	6 (31.6)		
Mosaic architecture				0.286			0.273	0.946
Absent	13 (36.1)	10 (22.7)	1.000		7 (43.8)	4 (21.1)		
Present	23 (63.9)	34 (77.3)	1.922 (0.722–5.119)		9 (56.2)	15 (78.9)		

HBP, hepatobiliary phase. Data are numbers of patients, with the percentage in parentheses. P-value with Chi-square test or Fisher exact test for categorical variables and Student t-test for numeric variables. ^*^Odds ratio (OR) with univariate test. ^†^p_Intra_: p-value between the MVI+ and MVI− groups. ^‡^p_Inter_: p-value between the training and validation cohorts.

### Construction and Validation of Radiomics Models

In spite of four classification algorithms applied, the highest AUCs were produced by LR or SVC. In contrast, the random forest or adaboost model demonstrated over-fitting of the AUC, approaching 1.00 in the training group and much higher than that in the validation group, suggesting that the classifiers were too volatile and unsuitable for classification in this study. The performance of the 24 single-sequence models using the LR or SVC classifier is shown in **Table 3**. Noticeably, single-sequence models based on T2WI, AP, and HBP showed satisfactory performance, with AUCs >0.75 in the training and validation datasets. Of these, the T2WI radiomics model in VOI_interface_ displayed optimal efficacy, with an AUC of 0.891 in the training group and 0.813 in the validation group, respectively. The HBP radiomics model in VOI_whole_ had an AUC of 0.862 in the training set and 0.806 in the validation set, showing comparable power with the T2WI radiomics model in VOI_interface_.

**Table 3:** The performance of single-sequence radiomics models based on different VOIs.

AUC (Training cohort/Validation cohort/Best classifier)	T2WI	T1WI	AP	PVP	DP	HBP
VOI_whole_	0.785	0.893	0.821	0.791	0.700	0.862
	0.618	0.714	0.776	0.622	0.691	0.806
	SVC	LR	LR	LR	SVC	LR
VOI_periphery_	0.857	0.897	0.757	0.850	0.819	0.736
	0.796	0.648	0.763	0.717	0.688	0.707
	LR	SVC	SVC	LR	SVC	SVC
VOI_whole + periphery_	0.824	0.763	0.846	0.776	0.900	0.799
	0.780	0.641	0.668	0.664	0.681	0.770
	LR	LR	SVC	LR	LR	LR
VOI_interface_	0.891	0.876	0.775	0.849	0.823	0.720
	0.813	0.618	0.776	0.628	0.704	0.701
	SVC	LR	SVC	LR	SVC	LR

AUC, area under the curve; VOI, the volume of interest; LR, logistic regression; SVC, support vector classifier; T2WI, T2-weighted imaging; T1WI, T1-weighted imaging; AP, arterial phase; PVP, portal venous phase; DP, delayed phase; HBP, hepatobiliary phase.

**Figure 4:**
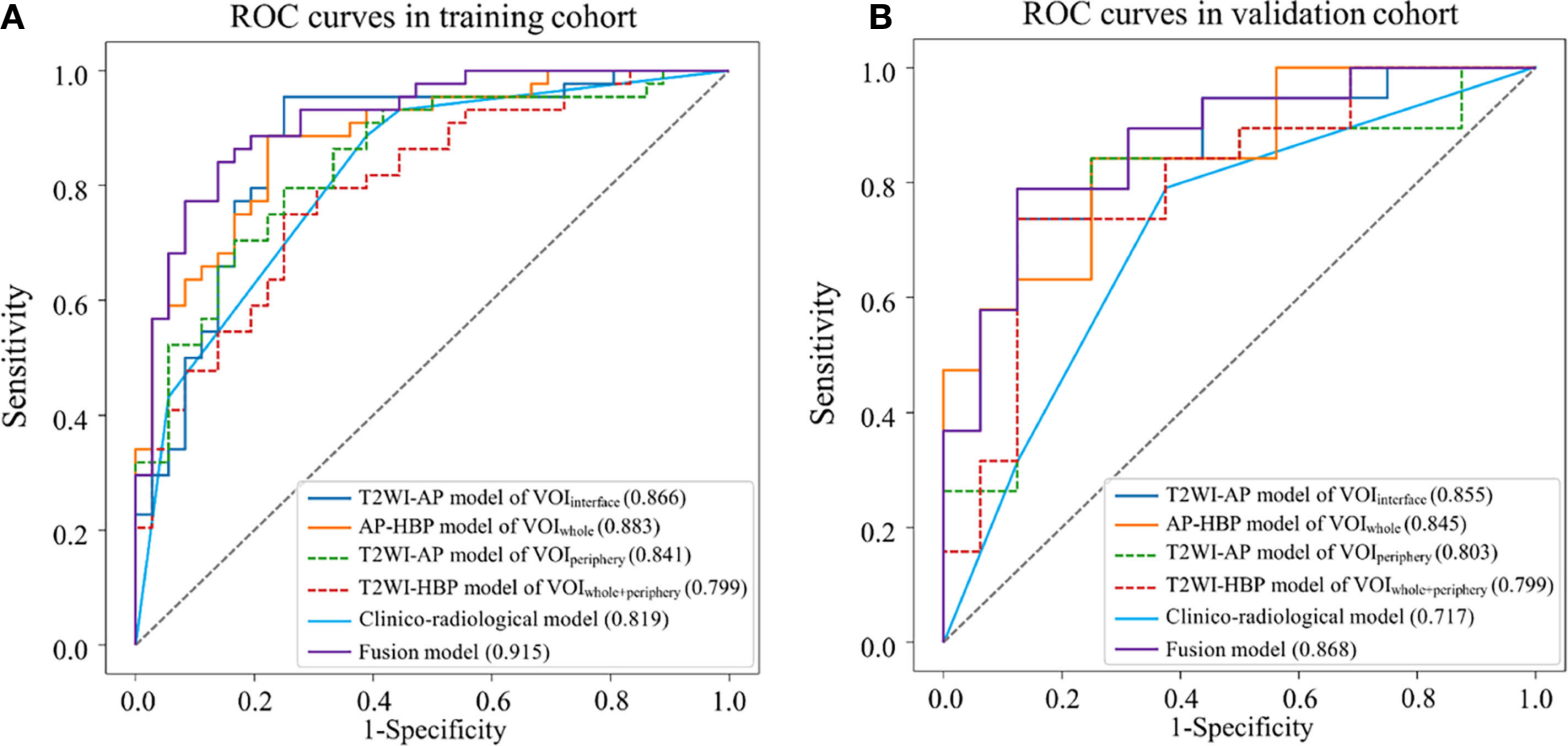
Receiver operating characteristic (ROC) curves of different models for predicting MVI. **(A)** training dataset; **(B)** validation dataset.

The multi-sequence radiomics models (**Table 4**, **Figures 4A, B**) were achieved with AUCs nearly or more than 0.8. T2WI-AP radiomics model in VOI_interface_ achieved AUCs of 0.866 (0.783–0.947) and 0.855 (0.731–0.963) for the training and validation cohorts, with highest accuracy of 0.863 and 0.800. The T2WI-AP radiomics signature in the VOI_interface_ was built using the logistic function to squeeze the output of a linear combination based on each selected feature and the corresponding coefficient between 0 and 1. **Table 5** shows the selected features and corresponding coefficients. In **Table S2**, three T2WI features were related to ‘nonsmooth margin’ (p <0.05). We found overfitting (AUC in training cohort higher than that in validation cohort) in the model using the classification algorithms except SVC (**Table S3**). Compared with the AUC of LR, that of SVC was higher in the validation cohort (**Table S4**). The detailed results of the four classifiers are shown in **Table S5, Figures S1, S2**.

**Table 4:** Predictive efficacy of multi-sequence radiomics models based on different VOIs.

VOIs	Best-sequence combination	Best classifier	Cohort	AUC (95% CI)	ACC	Sen	Spe	Thre^*^
VOI_whole_	AP + HBP	LR	TC	0.883 (0.801–0.948)	0.838	0.886	0.778	>0.510
			VC	0.845 (0.693–0.954)	0.743	0.842	0.625	
VOI_periphery_	T2WI + AP	SVC	TC	0.841 (0.753–0.922)	0.775	0.795	0.750	>0.271
			VC	0.803 (0.645–0.951)	0.800	0.737	0.875	
VOI_whole + periphery_	T2WI + HBP	LR	TC	0.799 (0.699–0.885)	0.750	0.750	0.750	>0.523
			VC	0.799 (0.643–0.941)	0.800	0.737	0.875	
VOI_interface_	T2WI + AP	SVC	TC	0.866 (0.783–0.947)	0.863	0.955	0.750	>0.537
			VC	0.855 (0.731–0.963)	0.800	0.842	0.750	

^*^Receiver operating characteristic analysis by maximizing the Youden index. VOI, the volume of interest; TC, training cohort; VC, validation cohort; AUC, area under the curve; CI, confidence interval; LR, logistic regression; SVC, support vector classifier. ACC, accuracy; Sen, sensitivity; Spe, specificity; Thre, threshold; T2WI, T2-weighted imaging; AP, arterial phase; HBP, hepatobiliary phase.

**Table 5:** Selected features in T2WI-AP radiomics model in VOI_interface_ and corresponding coefficients.

Radiomics features	Coefficients
AP original_glcm_InverseVariance	−1.26
AP original_glszm_SizeZoneNonUniformityNormalized	−0.84
AP original_glszm_ZoneVariance	1.09
T2 original_glcm_MaximumProbability	−2.07
T2 original_gldm_DependenceVariance	1.62
T2 original_gldm_LargeDependenceLowGrayLevelEmphasis	−1.40
T2 original_gldm_SmallDependenceLowGrayLevelEmphasis	1.66

Intercept = 0.13. T2WI, T2-weighted imaging; AP, arterial phase.

Besides, mean dice coefficients of all VOIs were nearly or higher than 0.75 (**Table S6**). The median values of interobserver ICC of radiomic features were ≥0.80 in different sequences or regions. The ICC was ≥0.8 for more than 85% of the features in VOI_whole_, VOI_whole + periphery_ and VOI_interface_, and was ≥0.7 for more than 80% of those in VOI _periphery_.

### Performance of the Fusion Model

**Figure 5:**
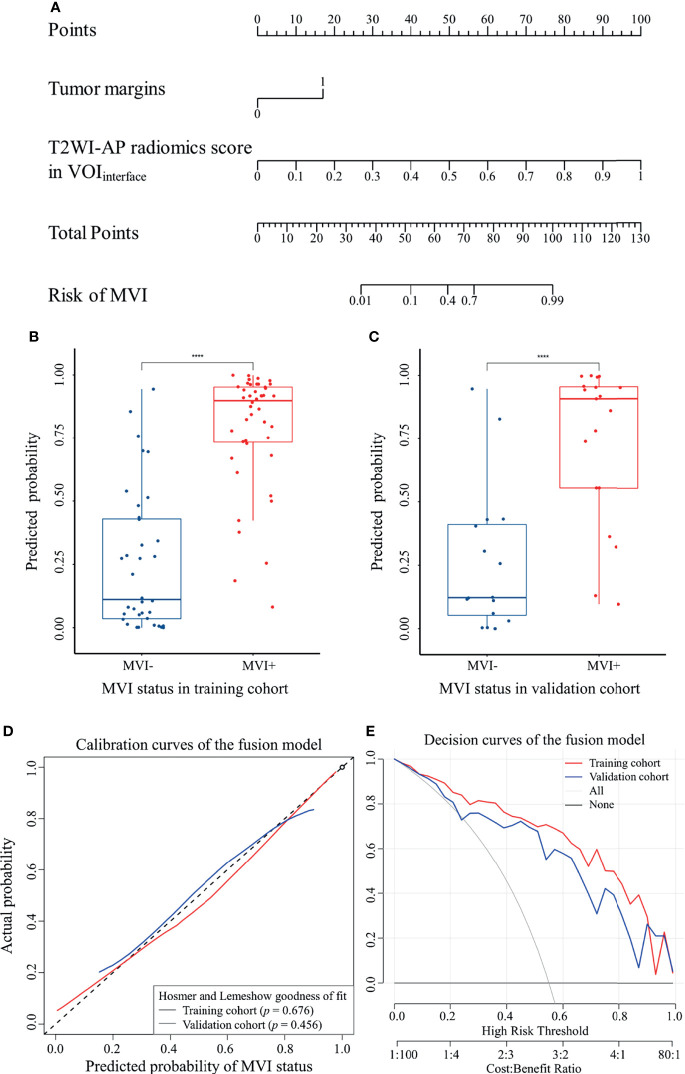
The fusion model of MVI was visualized as nomogram. **(A)** MVI nomogram; **(B)** and **(C)** box plot of MVI risk probabilities in the training cohort and validation cohort, ^****^ *p <* 0.0001 by Mann–Whitney U test; **(D)** calibration curves; **(E)** decision curves.

The fusion model was presented as a nomogram (**Figure 5A**) based on the combination of the T2WI-AP radiomics signature in VOI_interface_ and one radiological variable (tumor margin) by multivariate logistic regression (*p <*0.001), showing excellent prediction efficacy (AUC (95% CI) = 0.915 (0.853–0.976) and 0.868 (0.749–0.988) for training and validation cohorts, respectively). Box plot of MVI risk probabilities in the training cohort and validation cohort (**Figures 5B, C**) showed the statistical difference between MVI− and MVI+ groups. The accuracy, sensitivity, and specificity in the training and validation cohorts were 0.850, 0.841, 0.861, and 0.771, 0.684, 0.875, respectively, with a threshold of 0.576. AUCs were not statistically different compared to the T2WI-AP radiomics model in the VOI_interface_ (*p* = 0.097 in training set, *p* = 0.759 in validation set) but statistically different compared with the clinico-radiological model (*p* = 0.014 and 0.025, respectively).

IDI indicated a significant improvement in the predictive value of the fusion model compared to the T2WI-AP radiomics model (IDI (95% CI) = 0.333 (0.252–0.413) and 0.2544 (0.111–0.398) for the training and validation cohorts, respectively, *p <*0.05) and the clinico-radiological model (IDI (95% CI) = 0.191 (0.093–0.288) and 0.220 (0.066–0.374), respectively, *p <*0.05).The calibration curves (**Figure 5D**) of the nomogram showed that the model-predicted probability was well matched with the practical MVI status. Moreover, the decision curves (**Figure 5E**) demonstrated the clinical usefulness of the fusion model based on the net benefit of using the nomogram to predict MVI being greater than the assumption of all/none patients experiencing MVI.

## Discussion

This study aimed to investigate the predictive value of radiomics models for preoperative prediction of MVI status in HCC patients. Multi-region (VOI_whole_, VOI_periphery_, VOI_whole + periphery_, and VOI_interface_) radiomic models based on multi-sequence MRI were built and validated, as well as a clinico-radiological model constructed from clinical information and imaging data. The fusion model consisting of T2WI-AP radiomics signatures in the VOI_interface_ and the radiological predictor (non-smooth tumor margin) achieved a better discriminative efficacy than clinico-radiological model or the T2WI-AP radiomics model alone (IDIs >0).

Due to the peritumoral nature of MVI (25, 32), peritumoral areas in addition to the whole tumor were studied here. Radiomics models based on VOI_periphery_ and VOI_whole + periphery_ were predictive although not better than the models based on VOI_whole_ in discriminatory ability. Indeed, some studies have discussed the role of the tumor–liver interface as a plausible indicator of underlying distortion of tissue induced by MVI (23, 24, 33). Zheng et al. previously focused on the tumor–liver interface using CT quantitative image analysis; however, this study omitted the internal region of the tumor (23). Our study found that T2WI-AP radiomics models in the VOI_interface_ achieved an AUC over 0.75, in either the training or validation cohorts. Additionally, the selected features in the T2WI-AP model in VOI_interface_ (the optimal radiomics model) included two glcm features, three gldm features and two glszm features, textural features correlated with tumor heterogeneities, similar to the findings of Wilson et al. (34).

In this study, we also established single-sequence and multi-sequence radiomics models based on various lesion regions. The preponderance of VOI_whole + periphery_ over VOI_whole_ was not the same as the findings of Chong et al. (22), however both of our studies agree that the multi-sequence models outperformed the single-sequence models. HBP displayed the highest AUCs among the single-sequence models in VOI_whole_, in agreement with that of Yang et al. (19), but in contrast with the findings of Chong et al. (which found that PVP outperformed HBP) (22). Such disagreement may be related to disparities in MRI parameters or population characteristics.

In addition to radiomic analysis, conventional MRI features were assessed to predict MVI. Non-smooth tumor margin and peritumoral hypointensity on HBP were significant factors in the clinico-radiological model, with non-smooth tumor margin recognized as a predictor in the fusion model as well. Renzulli et al. proposed that MVI was more likely to occur when tumor margins were invaded (26), which resulted in non-smooth tumor margins. Peritumoral hypointensity on HBP associated with MVI may be rooted in peritumoral perfusion change influenced by decreased organic anion-transporting polypeptide expression caused by impaired hepatocytes (10, 35). Lee et al. additionally reported that non-smooth tumor margin, arterial peritumoral enhancement, and peritumoral hypointensity on HBP were independent predictors of MVI, but with specificity values of the combination of any two or all three radiologic indicators >90% and sensitivity values <55% (10). Feng et al. obtained similar results (36), concluding that radiomics models based on HBP are effective predictors of MVI compared to the radiological method. This finding was partly in line with our observation that the radiomics model had a higher AUC value than the clinico-radiological model, albeit without statistical significance.

Moreover, clinical factors were not significantly different between the MVI+ and MVI− groups in the training cohort according to univariate analysis in our study, except for AFP, despite statistical non-significance observed in AFP at multivariate analysis. This finding was not in accord with previous studies (20, 22) describing univariate and multivariate analyses in support of AFP as an effective factor, in contrast to findings from Zhang et al. (37).This may be due to differences in patient characteristics in some cases.

Some limitations should be noted in this study. First, the inherent selection biases in a single-center retrospective study with a limited sample size. To address this, we conducted cross-validation to optimize hyperparameters to avoid overfitting. Additionally, six MRI sequences were investigated using diverse classifiers for a more sufficient analysis. External validation by larger datasets from other centers is still needed. Additionally, VOIs were delineated manually or were merged or dilated automatically based on artificially drawn regions, which might lead to some VOI differences in various sequences. While some image processing was used to decrease the effect of heterogeneity in our study, in future studies, more automated and precise segmentation methods are required to improve the consistency and repeatability of the findings.

In conclusion, radiomics models are effective and noninvasive tools for preoperatively identifying MVI status. Here we present a fusion model incorporating a T2WI-AP radiomics signature in VOI_interface_ and non-smooth tumor margin as a potential biomarker for preoperative prediction of MVI, achieving desirable prediction of the individualized risk estimation of MVI in HCC patients.

## Data Availability Statement

The raw data supporting the conclusions of this article will be made available by the authors, without undue reservation.

## Ethics Statement

The studies involving human participants were reviewed and approved by The First Affiliated Hospital of Fujian Medical University. Written informed consent for participation was not required for this study in accordance with the national legislation and the institutional requirements.

## Author Contributions

Conception and design: YL, LG, and MX. Development of methodology: YL and LG. Acquisition of data: LG, MX, ZH, XC, CY, RY, and LZ. Analysis and interpretation of data: LG, MX, ZH, XC, and CY. Editing and review of the manuscript: all authors. Study supervision: YL. All authors listed have made a substantial, direct, and intellectual contribution to the work and approved it for publication.

## Funding

This study received funding by the Joint Funds for the Innovation of Science and Technology, Fujian Province (CN) (Award Number: 2019Y9125) and the startup fund for scientific research of Fujian Medical University (CN) (Award Number: 2019QH1097).

## Conflict of Interest

The authors declare that the research was conducted in the absence of any commercial or financial relationships that could be construed as a potential conflict of interest.

## Publisher’s Note

All claims expressed in this article are solely those of the authors and do not necessarily represent those of their affiliated organizations, or those of the publisher, the editors and the reviewers. Any product that may be evaluated in this article, or claim that may be made by its manufacturer, is not guaranteed or endorsed by the publisher.

## Abbreviations

AFP, Alpha-fetoprotein; AP, Arterial phase; AUC, Area under the curve; CT, Computed tomography; DP, Delayed phase; DWI, Diffusion-weighted imaging; GD-BOPTA, Dadobenate dimeglumine; HBP, Hepatobiliary phase; HCC, Hepatocellular carcinoma; ICC, intraclass correlation coefficient; IDI, Integrated discrimination improvement; INR, International normalized ratio; LR, Logistic Regression; MRI, Magnetic resonance imaging; MVI, Microvascular invasion; OR, Odds ratio; PVP, Portal venous phase; RFE, Recursive feature elimination; ROC, Receiver operating characteristic; SVC, Support Vector Classifier; T1WI, T1-weighted imaging; T2WI, T2-weighted imaging; VOIs, Volumes of interest.
