# Supplementary material for: Multi-Region Radiomic Analysis Based on Multi-Sequence MRI Can Preoperatively Predict Microvascular Invasion in Hepatocellular Carcinoma
Source: Front Oncol. 2022 Apr 27;12:818681. doi: 10.3389/fonc.2022.818681 (PMC9094629; doi:10.3389/fonc.2022.818681)
Supplement: Supplementary file 1 [file DataSheet_1.pdf]

Supplementary Materials

Contents

List of Supplementary Tables:

1. Table S1 MRI Acquisition Parameters.
2. Table S2 The correlationship between selected features in T2WI-AP radiomics model in VOIinterface and tumor margin.
3. Table S3 The comparison of AUCs between training and validation cohort of T2WI-AP model based on $VOI_{interface}$ in four classification algorithms using Delong test.
4. Table S4 The comparison of AUCs between T2WI-AP model based on $VOI_{interface}$ in four classification algorithms using Delong test.
5. Table S5 Results of best-sequence combinations using four algorithms.
6. Table S6 Mean dice coefficients of segmentation.

List of Supplementary Figure Legends:

1. Figure S1 The heatmap of AUCs in single-sequence radiomics model based on different regions using Adaboost (AB) or Random Forest (RF) algorithm.
2. Figure S2 The heatmap of AUCs in single-sequence radiomics model based on different regions using Logistic Regression (LR) or Support Vector Classifier (SVC) algorithm.

Supplementary Tables

Table S1 MRI Acquisition Parameters.

Sequence	Matrix	FOV (mm^2)	ST (mm)	Slice gap (mm)	TR (msec)	TE (msec)	Flip angle (degrees)
T2WI with fat suppression	168×320	21x38	5	1	2300	79	140
DWI (b=50, 800 sec/mm^2)	78×128	21x38	5	1	6000	73	-
In-phase and opposed-phase T1WI	192×256	21x38	5	1	133	1.23 and 2.46	70
VIBE-T1WI	182×320	25x38	3	0.6	3.9	1.4	9

Contrast agent: gadobenate dimeglumine (MultiHance; Bracco), at a dose of 0.2ml/kg and at a rate of 2mL/sec followed by a 20mL saline flush. T2WI, T2-weighted imaging; DWI, diffusion-weighted imaging; T1WI, T1-weighted imaging; VIBE, volumetric interpolated breath-hold examination; FOV, field of view; ST, section thickness; TR, repetition time; TE, echo time.

Table S2 The correlationship between selected features in T2WI-AP radiomics model in $VOI_{interface}$ and tumor margin.

Feature	ρ	*p*
AP_glcm_IV	0.056	0.554
AP_glszm_SZNUN	0.024	0.803
AP_glszm_ZV	0.083	0.380
T2WI_glcm_MP	-0.315	0.001
T2WI_gldm_DV	-0.013	0.894
T2WI_gldm_LDLGLE	-0.305	0.001
T2WI_gldm_SDLGLE	-0.288	0.002

Abbreviation: AP, arterial phase; T2WI, T2-weighted imaging; IV, InverseVariance; SZNUN,

SizeZoneNonUniformityNormalized; ZV, ZoneVariance; MP, Maximum-Probability; DV, Dependence-Variance; LDLGLE, LargeDependenceLowGray-LevelEmphasis; SDLGLE, SmallDependenceLowGrayLevelEmphasis.

Table S3 The comparison of AUCs between training and validation cohort of T2WI-AP model based on $VOI_{interface}$ in four classification algorithms using Delong test.

Algorithms	Cohort	AUCs	*p*
AB	Train vs. Validation	1.000 vs. 0.763	0.007
LR	Train vs. Validation	0.917 vs. 0.711	0.034
RF	Train vs. Validation	1.000 vs. 0.747	0.008
SVC	Train vs. Validation	0.866 vs.0.855	0.751

Abbreviation: AB, Adaboost; LR, Logistic Regression; RF, Random Forest; SVC, Support Vector Classifier; AUC, area under the curve; T2WI, T2-weighted imaging; AP, Arterial phase.

Table S4 The comparison of AUCs between T2WI-AP model based on $VOI_{interface}$ in four classification algorithms using Delong test.

Algorithms	Cohort	AUCs	*p*
SVC vs. AB	Train	0.866 vs. 1.000	<0.001
	Validation	0.855 vs. 0.763	0.602
SVC vs. LR	Train	0.866 vs. 0.917	0.077
	Validation	0.855 vs. 0.711	0.045
SVC vs. RF	Train	0.866 vs. 1.000	<0.001
	Validation	0.855 vs. 0.747	0.53
LR vs. AB	Train	0.917 vs. 1.000	0.004
	Validation	0.711 vs. 0.763	0.572
LR vs. RF	Train	0.917 vs. 1.000	0.004
	Validation	0.711 vs. 0.747	0.743

AB vs. RF	Train	1.000 vs. 1.000	1.000
	Validation	0.763 vs. 0.747	0.821

Abbreviation: AB, Adaboost; LR, Logistic Regression; RF, Random Forest; SVC, Support Vector Classifier; AUC, area under the curve; T2WI, T2-weighted imaging; AP, Arterial phase.

Table S5 Results of best-sequence combinations using four algorithms.

VOIs	Best-sequence combination	Classifier	AUC (TC/VC)	ACC (TC/VC)
VOI_{whole}	AP + HBP	LR	0.883/0.845	0.838/0.743
		SVC	0.891/0.770	0.838/0.686
		AB	0.991/0.786	0.963/0.686
		RF	1.000/0.711	1.000/0.686
$VOI_{periphery}$	T2WI + AP	LR	0.859/0.720	0.800/0.657
		SVC	0.841/0.803	0.775/0.800
		AB	1.000/0.697	1.000/0.629
		RF	1.000/0.628	1.000/0.629
$VOI_{whole+periphery}$	T2WI + HBP	LR	0.799/0.799	0.750/0.800
		SVC	0.694/0.813	0.700/0.714
		AB	0.965/0.605	0.900/0.571
		RF	0.998/0.831	0.975/0.743
$VOI_{interface}$	T2WI + AP	LR	0.917/0.711	0.850/0.600
		SVC	0.866/0.855	0.863/0.800
		AB	1.000/0.763	1.000/0.686
		RF	1.000/0.747	1.000/0.686

Abbreviation: AB, Adaboost; LR, Logistic Regression; RF, Random Forest; SVC, Support Vector Classifier; AUC, area under the curve; T2WI, T2-weighted imaging; AP, Arterial phase; HBP, Hepatobiliary phase; TC, Training cohort; VC, Validation cohort.

Table S6 Mean dice coefficients of segmentation.

VOI	T2WI	T1WI	AP	PVP	DP	HBP
VOI_{whole}	0.944	0.947	0.957	0.950	0.944	0.959
$VOI_{periphery}$	0.746	0.769	0.791	0.767	0.764	0.812
$VOI_{whole+periphery}$	0.940	0.943	0.953	0.941	0.941	0.961
$VOI_{Interface}$	0.837	0.835	0.857	0.847	0.830	0.861

Abbreviation: T2WI, T2-weighted imaging; T1WI, T1-weighted imaging; AP, Arterial phase; PVP, Portal venous phase; DP, Delayed phase; HBP, Hepatobiliary phase.

Supplementary Figure Legends

Figure S1 The heatmap of AUCs in single-sequence radiomics model based on different regions using Adaboost (AB) or Random Forest (RF) algorithm.

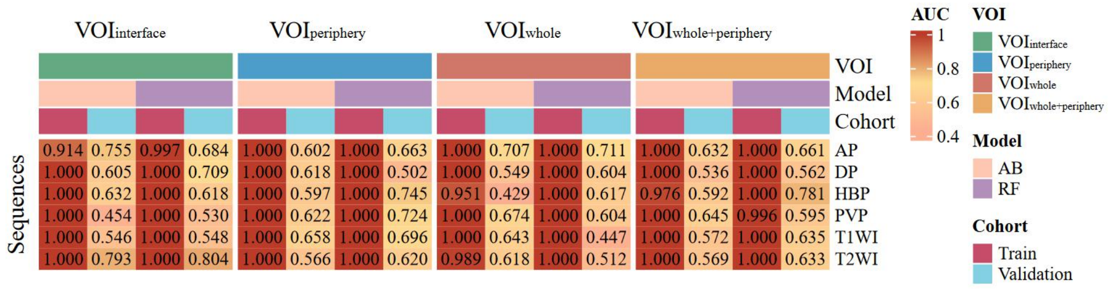

Abbreviation: AP, arterial phase; IV, InverseVariance; SZNUN, SizeZoneNonUniformityNormalized; ZV, ZoneVariance; MP, MaximumProbability; DV, Dependence-Variance; LDLGLE, LargeDependenceLowGrayLevelEmphasis; SDLGLE, SmallDependenceLow-GrayLevelEmphasis.

Figure S2 The heatmap of AUCs in single-sequence radiomics model based on different regions using Logistic Regression (LR) or Support Vector Classifier (SVC) algorithm.

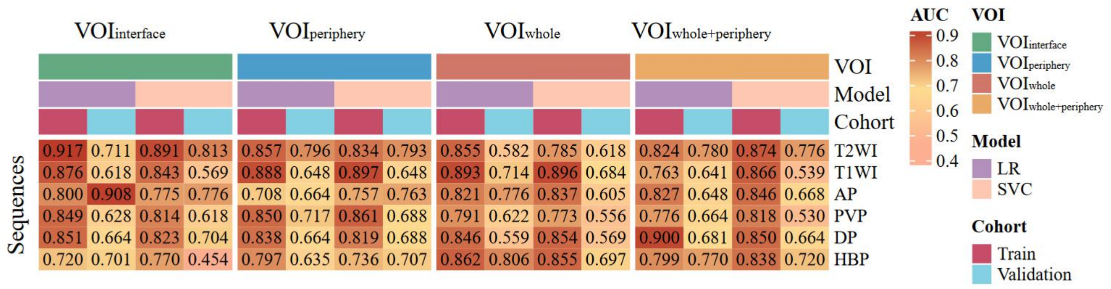

Abbreviation: AP, arterial phase; IV, InverseVariance; SZNUN, SizeZoneNonUniformityNormalized; ZV, ZoneVariance; MP, MaximumProbability; DV,

DependenceVariance; LDLGLE, LargeDependenceLowGrayLevelEmphasis; SDLGLE, SmallDependenceLow-GrayLevelEmphasis.
